# Interventions towards barriers to the practice of physical activity in adolescence: A systematic review protocol

**DOI:** 10.1371/journal.pone.0287868

**Published:** 2023-07-12

**Authors:** Lauryane Fonseca Terra, Woska Pires da Costa, Regina Márcia Ferreira Silva, Leonardo Mateus Teixeira de Rezende, Matias Noll, Priscilla Rayanne E. Silva Noll

**Affiliations:** 1 Instituto Federal Goiano—Campus Ceres, Ceres, Goiás, Brazil; 2 Instituto Federal Goiano—Campus Morrinhos, Morrinhos, Goiás, Brazil; 3 Instituto Federal de Goiás—Campus Itumbiara, Itumbiara, Goiás, Brazil; 4 Universidade Federal de Goiás, Goiânia, Goiás, Brazil; 5 Faculdade de Medicina, Universidade de São Paulo, São Paulo, Brazil; University of Toronto Temerty Faculty of Medicine, CANADA

## Abstract

**Introduction:**

Lack of regular physical activity is recognized as a global public health issue. Three out of every four adolescents do not adhere to physical activity recommendations. Thus, this systematic review will evaluate interventions employed to minimize barriers to physical activity among adolescents. Herein, we present the study protocol. To the best of our knowledge, this will be the first systematic review to assess the interventions implemented to minimize barriers to the practice of physical activity among adolescents. A systematic understanding of the most effective interventions to reduce the barriers to physical activity is essential.

**Method and analysis:**

We will search five databases: two multidisciplinary (Scopus and Web of Science) and three other health-related databases (Embase, SPORTDiscus, and PubMed). The search will be limited to original peer-reviewed articles published in English, with no time restrictions. The search strategy will use MeSH terms and their variations to maximize the search strategy. Two reviewers will independently read the included articles, extract the data, and evaluate the methodological quality using the Grading of Recommendations, Assessment, Development, and Evaluation scale and the risk of bias using the Critical Appraisal Skills Programme checklist and Downs and Black scale. Discrepancies will be resolved by a third reviewer. This systematic review will follow the guidelines outlined in the 2020 Preferred Reporting Items for Systematic Reviews and Meta-Analyses.

**Discussion:**

The outcomes of this study are expected to enhance the current understanding of the obstacles to physical activity among adolescents and aid in the development or modification of programs to combat physical inactivity in this population. Consequently, these findings should have a positive impact on current and future adolescent health outcomes.

**Ethics and disclosure:**

Ethical approval will not be required for this study as it is an analysis of previously published articles (i.e., secondary data). The results will be published in a peer-reviewed journal.

**PROSPERO registration:** CRD42022382174.

## Introduction

Regular physical activity (PA) is known to promote health, enhance quality of life, and have a positive impact on several social and behavioral aspects [1–3]. PA contributes to various health benefits such as cardiorespiratory, metabolic, musculoskeletal, functional, cancer prevention, several other non-communicable chronic diseases (NCDs), and mental health [3–5]. In contrast, the absence of PA is associated with the development of NCDs and increased mortality and is considered one of the main behavioral risk factors for current and future adolescent health outcomes [6, 7]. Particularly concerning is the prevalence of physical inactivity among adolescents, as three of four individuals are inactive [8].

Adolescents may encounter obstacles or barriers that prevent them from achieving the recommended number of regular weekly activities necessary for good health and wellbeing [3]. Barriers are a set of factors that hinder the attainment of a particular goal [9]. These barriers are linked to several sociodemographic, biological, psychological, cognitive, environmental, and behavioral dimensions [10]. Previous studies have identified several barriers to regular PA among adolescents, including laziness, lack of company, lack of time, lack of willingness, and difficulty in accessing facilities [10–13].

Given the prevalence of physical inactivity among adolescents [8, 14], establishing efficient means to increase the practice of PA is critical. Interventions that aim to promote regular PA among adolescents involve developing or replicating projects, programs, and actions that reduce inactivity [15, 16]. These strategies are segmented and promising because they influence the behavioral activities of this population [15, 17]. However, recent studies have pointed out the lack of interventions that specifically address the barriers preventing adolescents from engaging in PA [18, 19]. Systematizing results on the most effective interventions to minimize these barriers, despite the scarcity of existing intervention studies, is essential.

Systematically understanding interventions and their contexts concerning barriers to PA is crucial. In this context, we will highlight the following research questions: "What interventions are performed to reduce barriers to PA?" and "Among the existing barriers, which are most significant?" To the best of our knowledge, this will be the first systematic review to evaluate interventions implemented to minimize barriers to the practice of PA among adolescents. These results may enhance the scientific understanding of the topic and propose more effective interventions. In addition, it can aid institutional managers and encourage the academic community to develop or replicate projects, programs, and actions that promote regular PA among adolescents.

## Method

### Protocol and registration

This systematic review protocol was prepared in compliance with the Preferred Reporting Items for Systematic Reviews and Meta-Analyses (PRISMA) for Protocols 2015 (PRISMA-P 2015), as applicable (S1 File) [20–22]. The PRISMA-P 2015 comprises a checklist of several items designed to aid in the preparation and development of a systematic review protocol [20]. After defining the research question, a preliminary database search was conducted to identify studies that support the development of this protocol [23].

This protocol is currently registered in the International Prospective Register of Systematic Reviews (PROSPERO) (registration number: CRD42022382174). Any necessary modifications to this protocol will be made during the execution of the study. If any changes occur, they will be reported to PROSPERO and explicitly stated in the final version of the article prior to publication.

### Search strategy and databases

A systematic review will be conducted to gather bibliographic data from the following databases: Web of Science Core Collection^*™*^, Scopus^™^, MEDLINE/PubMed^®^ via the National Library of Medicine^®^ interface, Embase^™^, and SPORTDiscus^®^ via the EBSCOhost^™^ interface. Databases will be searched in July 2023 to identify potential articles for inclusion in this systematic review. Additionally, the search strategy will be complemented by screening the reference lists of the included studies and relevant systematic reviews in the subsequent months.

This systematic review will employ the following Medical Subject Headings (MeSH) terms as the primary search terms: "intervention," "barrier," "physical activity," and "adolescent." The Boolean operator “OR” will be used to group the synonyms of each term. The terms will then be organized into blocks and connected by the Boolean operator "AND" to complete the search strategy (Table 1). Adaptations of the MeSH terms and their respective synonyms will be made to account for the specificity of each database to be searched (S2 File). The titles, abstracts, and keyword fields of the databases will be searched.

**Table 1:** Keywords used in the search strategy organized in to blocks.

Blocks (PICO)	
#1	"adolescent" OR "adolescents" OR "adolescence" OR "teen" OR "teens" OR "teenager" OR "teenagers" OR "youth" OR "young"
P	
#2	"intervention" OR "interventions" OR "action" OR "actions" OR "program" OR "programs" OR "health education" OR "primary prevention" OR "health promotion" OR "primary health care"
I	
#3	"barrier" OR "barriers" OR "obstacle" OR "obstacles" OR "challenge" OR "challenges" OR "difficulty" OR "difficulties" OR "facility access"
C	
#4	"physical activity" OR "physical activities" OR "physical inactivity" OR "sedentary lifestyle" OR "sedentary behavior" OR "sedentary time" OR "exercise" OR "exercises"
O	
**Search string**:	(#1) AND (#2) AND (#3) AND (#4)

**Note:** PICO stands for Population, Intervention, Comparison, Outcome; furthermore, it is a framework created to define the issue addressed by the systematic review.

The metadata extraction from the databases will adhere to the guidelines presented in the PRISMA-Search checklist (PRISMA-S) [24]. This checklist is an extension of the PRISMA and encompasses several aspects of the literature search process for systematic reviews. It covers topics such as the specificities of the selected databases, search strategy (including the registration of limits, restrictions, and filters), and the process of documenting the returned records and deduplication [21].

### Eligibility criteria

The eligibility criteria for this study were determined using the Population, Intervention, Comparison, Outcome (PICO) [25–27] search strategy for evidence-based health research. Accordingly, the following parameters were established: population (P), both male and female adolescents aged 10–19 years; intervention (I), any actions and programs aimed at reducing barriers to PA; comparison (C), absence of interventions; and outcome (O), practice of PA.

This systematic review will include relevant articles published in English without date restrictions. To be considered eligible, articles must meet the following inclusion criteria. However, articles will be excluded if they meet at least one of the previously defined exclusion criteria. The validity of the eligible studies will be evaluated, and any records of retraction will be identified using the Scite tool [24].

Inclusion criteria:

- Original published and peer-reviewed studies.
- Studies written in English.
- Studies focusing on interventions aimed at reducing barriers to PA in adolescents.
- Studies including adolescents aged 10–19 years, according to the World Health Organization definition [3].

Exclusion criteria:

- Duplicates: Studies published in more than one journal will be carefully reviewed to confirm elimination [28].
- Studies involving age groups other than adolescents unless the data are reported separately or can be calculated from the data provided.
- Studies including adolescents with physical or mental disabilities or who presented with chronic diseases during sampling.
- Studies of interventions conducted in clinical settings and/or nursing homes.
- Studies on interventions conducted in specific populations, such as rural, indigenous, refugee, and/or isolated groups.
- Studies with incomplete data, opinion articles, case reports, comments, editorials, dissertations, theses, reviews, and cross-sectional studies.
- Studies that are not accessible even after attempts to contact the authors.
- Studies that include retractions [24].

### Review process

After implementing the search strategy, the information related to the identified articles will be extracted and imported using EndNote^*™*^ X9 software (Clarivate, Philadelphia, PA, USA) to eliminate any duplicates [28]. Subsequently, the metadata will be transferred using Rayyan software (Rayyan Systems Inc., Cambridge, MA, USA), where it will be evaluated by the reviewers [24] after activating the blinding feature. Rayyan is a software tool designed to aid researchers conducting systematic reviews and meta-analyses [29].

The first stage of the review process will involve the full reading and selection of titles and abstracts by two independent reviewers (LFT and LMTR) [24, 30, 31]. Any disagreements will be resolved by one of the senior researchers (PRSN or MN) [30–32]. Eligibility will be determined based on the inclusion and exclusion criteria. The percentage agreement and Cohen’s kappa coefficients will be calculated [33] to evaluate the reliability of the reviewers’ evaluations.

**Fig 1:**
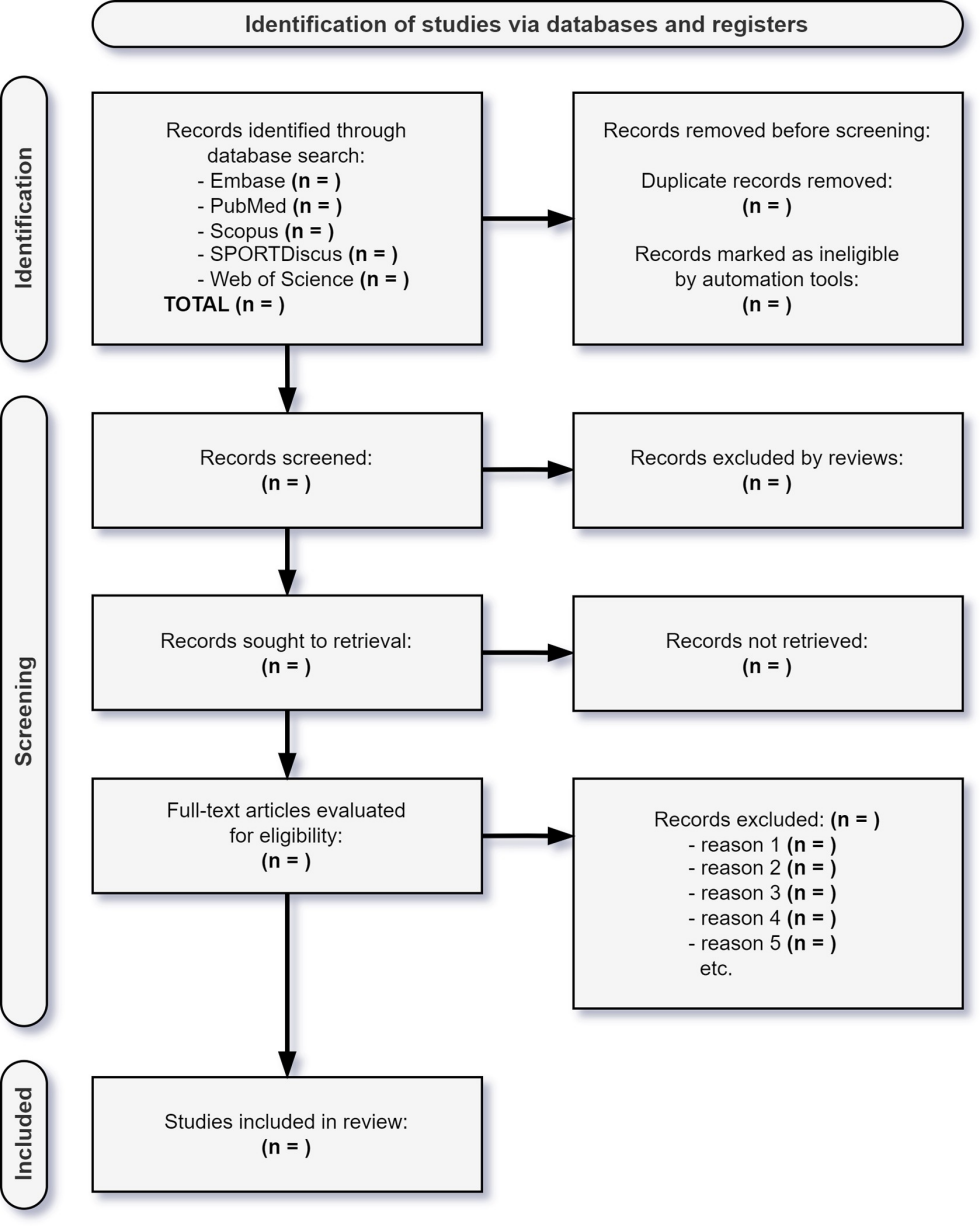
PRISMA 2020 flow diagram for the identification, screening, and inclusion of studies in this review [32].

In the second stage of the review process, two independent reviewers (LFT and LMTR) will analyze the full texts of the articles selected in the first stage, and any disagreements will be resolved by consensus with a third researcher (PRSN or MN). Finally, the articles that meet the inclusion criteria will be included in the systematic review. The selection process will be illustrated in a flowchart (Fig 1).

### Data extraction, synthesis, and analysis

A spreadsheet that includes all aspects described in Table 2 will be used for data extraction. Two independent reviewers (LFT and LMTR) will evaluate and extract the data. Any discrepancies will be addressed by consensus with a third reviewer (PRSN or MN).

**Table 2:** Main version of the table used to extract data from the included studies.

#	Author, Year, and Country of publication	Study design/Type	Intervention Used	Sample	Period in which data collection occurred	Type of study (quali/quanti/mixed)	Instrument used for data collection	Type of analysis performed	Main results	Gaps found
**1**										
**2**										
**…**										
**Hypothetical example**	COSTA 2022 Spain	• Experimental • Longitudinal • Prospective • Analytic • Comparative • Case-control	Playful activities and interclass competitions.	253 high school students	2018–2019	Qualitative	Questionnaire	Chi-square test	• Playful activities that encourage students to practice swimming.	• Analyzed the public educational institutions regarding the infrastructure conditions for the practice of swimming by students. • Analyzed the educational institutions regarding the staff of professionals focused on the practices of activities and profiles.
**…**										
***n***										

We will conduct descriptive and content analyses of the results based on the intervention attributes related to PA to identify the factors to be examined in this study [34]. In addition, we will compare the methods and effectiveness of each intervention. Descriptive analysis of the included articles will be conducted to identify the factors under study. In cases of missing relevant data, we will attempt to contact the authors [35].

### Assessment of quality and risk of bias

The methodological quality of the evidence will be assessed using the Grading of Recommendations, Assessment, Development, and Evaluation (GRADE) approach [36–38]. GRADEpro GDT software (McMaster University and Evidence Prime, Inc., Hamilton, ON, Canada) will be used to classify the studies as high quality, moderate quality, low quality, or very low quality [37].

The risk of bias in the included qualitative studies will be assessed using the Critical Appraisal Skills Programme checklist [39, 40]. Each study will be scored according to three categories: a) low quality (one star; 0–3 points), b) moderate quality (two stars; 4–7 points), and c) high quality (three stars; 8–10 points) [39, 41].

Quantitative studies will be evaluated using the 27-item Downs and Black scale [41]. For observational studies in which some items on this scale are not applicable, an adapted version of the scale will be used for both cross-sectional (0–12 points) and longitudinal (0–16 points) studies [13, 30, 37]. A quality score will be calculated for each study; the obtained score will be expressed as a percentage relative to the maximum possible score for the study design [35]. In summary, studies with scores equal to or above 70% will be considered to have a low risk of bias [42].

Two independent reviewers (LFT and LMTR) will evaluate the methodological quality and risk of bias [35]. In the event of any disagreement in the evaluations made by the two reviewers, a third reviewer (PRSN or MN) will be consulted [35].

### Reviewer training

The researchers responsible for determining the eligibility of articles will receive training on the use of the inclusion and exclusion criteria [30, 43, 44]. In addition, they will undergo preparatory training on how to apply the instruments to evaluate the methodological quality and risk of bias based on reading scientific text abstracts [30].

## Discussion

Regular PA is crucial for promoting health and quality of life while reducing NCDs [7, 45]. However, PA remains a persistent problem among adolescents [7, 8], with frequently cited barriers including lack of time, support, motivation, and adequate environments, and laziness [9, 10, 13, 14]. The aim of the study is to identify original articles that implemented interventions to address these barriers and reduce physical inactivity in adolescents. Given the global mortality rate attributed to NCDs [7, 45], evaluating group interventions capable of mitigating this public health problem is essential to promote adolescent health.

Strengths and limitations

This systematic review has several strengths that make it a pioneer in this field. First, it facilitates a systematic evaluation of the interventions used to minimize barriers to PA among adolescents, which may inform the development and improvement of programs aimed at reducing the physical inactivity. In addition, because it is a scarce topic, this protocol addresses the use of broad techniques and terms in the search strategy after validation. Notably, the search will be conducted in four databases without time restrictions to better identify studies on the subject. In addition, through the application of methods already employed and validated in other studies, the results are expected to aid the scientific community and fill the gaps in the literature. Therefore, we intend to publish the results of this systematic review in specialized journals in the field to share our findings and expand the reach of professionals and interests in the area.

One potential limitation of systematic reviews is the possibility that no study meets the predetermined eligibility criteria, resulting in an "empty" review. However, even if this occurs, the review may still provide value to the scientific community by promoting further research on the topic and encouraging efforts to address research problems [46]. Owing to the pioneering nature of this topic, we expect that many studies will be conducted on interventions to minimize barriers to PA among adolescents, potentially because of the time constraints in developing strategies [47]. Another limitation of this review may be related to its focus solely on the adolescent population, which may restrict the analysis of the results. Therefore, acknowledging these possible limitations that may make the analysis of the results challenging or even unfeasible is important.

## Conclusion

This study highlights the global prevalence of PA among adolescents, which has significant implications for public health. To address this issue, it is essential to promote PA activity early in life. However, multiple barriers must be considered as influential factors in various psychological, sociocultural, emotional, environmental, and sociodemographic dimensions [47]. Therefore, the aim of this study is to gather scientific evidence by analyzing previously published articles, which will allow us to evaluate the interventions aimed at minimizing these barriers. Ultimately, the resulting data and conclusions can assist managers and the academic community in developing and implementing more effective interventions to promote PA among adolescents.

## Supporting information

S1 File The PRISMA-P checklist for this systematic review protocol. (PDF)

S2 File Details of Boolean search string used for each database. (PDF)
