# Supplementary material for: Interventions towards barriers to the practice of physical activity in adolescence: A systematic review protocol
Source: PLoS One. 2023 Jul 12;18(7):e0287868. doi: 10.1371/journal.pone.0287868 (PMC10337968; doi:10.1371/journal.pone.0287868)
Supplement: S2 File — (PDF) [file pone.0287868.s002.pdf]

Appendix S2

Details of Boolean search string for each database.

Web of Science™ (WoS) Core Collection via native interface	
**Blocks and Returns**	**Search strings**
#1 (Return: ‹n_1›)	TS=("adolescent" OR "adolescents" OR "adolescence" OR "teen" OR "teens" OR "teenager" OR "teenagers" OR "youth" OR "young")
#2 (Return: ‹n_2›)	TS=("intervention" OR "interventions" OR "action" OR "actions" OR "program" OR "programs" OR "health education" OR "primary prevention" OR "health promotion" OR "primary health care")
#3 (Return: ‹n_3›)	TS=("barrier" OR "barriers" OR "obstacle" OR "obstacles" OR "challenge" OR "challenges" OR "difficulty" OR "difficulties" OR "facility access")
#4 (Return: ‹n_4›)	TS=("physical activity" OR "physical activities" OR "physical inactivity" OR "sedentary lifestyle" OR "sedentary behavior" OR "sedentary time" OR "exercise" OR "exercises")
#5 (Return: ‹n_5›)	(#1) AND (#2) AND (#3) AND (#4)
	**Search refinement**:<br>1. Document type: Article<br>2. Language: English<br>**Return after refinement**:<br>‹*N*› studies in the test carried out in ‹*date*›.

Scopus™ via native interface	
**Blocks and Returns**	**Search strings**
#1<br>(Return: ‹n_1›)	TITLE-ABS-KEY ("adolescent" OR "adolescents" OR "adolescence" OR "teen" OR "teens" OR "teenager" OR "teenagers" OR "youth" OR "young")
#2<br>(Return: ‹n_2›)	TITLE-ABS-KEY ("intervention" OR "interventions" OR "action" OR "actions" OR "program" OR "programs" OR "health education" OR "primary prevention" OR "health promotion" OR "primary health care")
#3<br>(Return: ‹n_3›)	TITLE-ABS-KEY ("barrier" OR "barriers" OR "obstacle" OR "obstacles" OR "challenge" OR "challenges" OR "difficulty" OR "difficulties" OR "facility access")
#4<br>(Return: ‹n_4›)	TITLE-ABS-KEY ("physical activity" OR "physical activities" OR "physical inactivity" OR "sedentary lifestyle" OR "sedentary behavior" OR "sedentary time" OR "exercise" OR "exercises")
#5<br>(Return: ‹n_5›)	(#1) AND (#2) AND (#3) AND (#4)
	**Search refinement**:<br>1. Document type: Article<br>2. Language: English<br>**Return after refinement**:<br>‹*N*› studies in the test carried out in ‹*date*›.

MEDLINE/PubMed® via interface of the National Library of Medicine® (NLM®)	
**Blocks and Returns**	**Search strings**
#1<br>(Return: ‹n_1›)	"adolescent"[Title/Abstract] OR "adolescents"[Title/Abstract] OR "adolescence"[Title/Abstract] OR "teen"[Title/Abstract] OR "teens"[Title/Abstract] OR "teenager"[Title/Abstract] OR "teenagers"[Title/Abstract] OR "youth"[Title/Abstract] OR "young"[Title/Abstract]
#2<br>(Return: ‹n_2›)	"intervention"[Title/Abstract] OR "interventions"[Title/Abstract] OR "action"[Title/Abstract] OR "actions"[Title/Abstract] OR "program"[Title/Abstract] OR "programs"[Title/Abstract] OR "health education"[Title/Abstract] OR "primary prevention"[Title/Abstract] OR "health promotion"[Title/Abstract] OR "primary health care"[Title/Abstract]
#3<br>(Return: ‹n_3›)	"barrier"[Title/Abstract] OR "barriers"[Title/Abstract] OR "obstacle"[Title/Abstract] OR "obstacles"[Title/Abstract] OR "challenge"[Title/Abstract] OR "challenges"[Title/Abstract] OR "difficulty"[Title/Abstract] OR "difficulties"[Title/Abstract] OR "facility access"[Title/Abstract]
#4<br>(Return: ‹n_4›)	"physical activity"[Title/Abstract] OR "physical activities"[Title/Abstract] OR "physical inactivity"[Title/Abstract] OR "sedentary lifestyle"[Title/Abstract] OR "sedentary behavior"[Title/Abstract] OR "sedentary time"[Title/Abstract] OR "exercise"[Title/Abstract] OR "exercises"[Title/Abstract]
#5<br>(Return: ‹n_5›)	(#1) AND (#2) AND (#3) AND (#4)
	**Search refinement**:<br>1. Language: English<br>**Return after refinement**:<br>‹*N*› studies in the test carried out in ‹*date*›.

Embase™ via native interface

**Blocks and Returns**	**Search strings**
#1<br>(Return: ‹n_1›)	'adolescent':ti,ab,kw OR 'adolescents':ti,ab,kw OR 'adolescence':ti,ab,kw OR 'teen':ti,ab,kw OR 'teens':ti,ab,kw OR 'teenager':ti,ab,kw OR 'teenagers':ti,ab,kw OR 'youth':ti,ab,kw OR 'young':ti,ab,kw
#2<br>(Return: ‹n_2›)	'intervention':ti,ab,kw OR 'interventions':ti,ab,kw OR 'action':ti,ab,kw OR 'actions':ti,ab,kw OR 'program':ti,ab,kw OR 'programs':ti,ab,kw OR 'health education':ti,ab,kw OR 'primary prevention':ti,ab,kw OR 'health promotion':ti,ab,kw OR 'primary health care':ti,ab,kw
#3<br>(Return: ‹n_3›)	'barrier':ti,ab,kw OR 'barriers':ti,ab,kw OR 'obstacle':ti,ab,kw OR 'obstacles':ti,ab,kw OR 'challenge':ti,ab,kw OR 'challenges':ti,ab,kw OR 'difficulty':ti,ab,kw OR 'difficulties':ti,ab,kw OR 'facility access':ti,ab,kw
#4<br>(Return: ‹n_4›)	'physical activity':ti,ab,kw OR 'physical activities':ti,ab,kw OR 'physical inactivity':ti,ab,kw OR 'sedentary lifestyle':ti,ab,kw OR 'sedentary behavior':ti,ab,kw OR 'sedentary time':ti,ab,kw OR 'exercise':ti,ab,kw OR 'exercises':ti,ab,kw
#5<br>(Return: ‹n_5›)	(#1) AND (#2) AND (#3) AND (#4)
	**Search refinement**:<br>1. Language: English<br>**Return after refinement**:<br>‹*N*› studies in the test carried out in ‹*date*›.

SPORTDiscus® via EBSCOhost™ interface	
**Blocks and Returns**	**Search strings**
#1<br>(Return: ‹n_1›)	TI("adolescent" OR "adolescents" OR "adolescence" OR "teen" OR "teens" OR "teenager" OR "teenagers" OR "youth" OR "young") **OR** AB("adolescent" OR "adolescents" OR "adolescence" OR "teen" OR "teens" OR "teenager" OR "teenagers" OR "youth" OR "young") **OR** MW("adolescent" OR "adolescents" OR "adolescence" OR "teen" OR "teens" OR "teenager" OR "teenagers" OR "youth" OR "young")
#2<br>(Return: ‹n_2›)	TI("intervention" OR "interventions" OR "action" OR "actions" OR "program" OR "programs" OR "health education" OR "primary prevention" OR "health promotion" OR "primary health care") **OR** AB("intervention" OR "interventions" OR "action" OR "actions" OR "program" OR "programs" OR "health education" OR "primary prevention" OR "health promotion" OR "primary health care") **OR** MW("intervention" OR "interventions" OR "action" OR "actions" OR "program" OR "programs" OR "health education" OR "primary prevention" OR "health promotion" OR "primary health care")
#3<br>(Return: ‹n_3›)	TI("barrier" OR "barriers" OR "obstacle" OR "obstacles" OR "challenge" OR "challenges" OR "difficulty" OR "difficulties" OR "facility access") **OR** AB("barrier" OR "barriers" OR "obstacle" OR "obstacles" OR "challenge" OR "challenges" OR "difficulty" OR "difficulties" OR "facility access") **OR** MW("barrier" OR "barriers" OR "obstacle" OR "obstacles" OR "challenge" OR "challenges" OR "difficulty" OR "difficulties" OR "facility access")
#4<br>(Return: ‹n_4›)	TI("physical activity" OR "physical activities" OR "physical inactivity" OR "sedentary lifestyle" OR "sedentary behavior" OR "sedentary time" OR "exercise" OR "exercises") **OR** AB("physical activity" OR "physical activities" OR "physical inactivity" OR "sedentary lifestyle" OR "sedentary behavior" OR "sedentary time" OR "exercise" OR "exercises") **OR** MW("physical activity" OR "physical activities" OR "physical inactivity" OR "sedentary lifestyle" OR "sedentary behavior" OR "sedentary time" OR "exercise" OR "exercises")
#5<br>(Return: ‹n_5›)	(#1) AND (#2) AND (#3) AND (#4)
	**Search refinement**:<br>1. Search Expanders: Apply related words and Apply equivalent subjects<br>1. Types of documents: Academic journals<br>**Return after refinement**:<br>‹*N*› studies in the test carried out in ‹*date*›.

Note: EBSCO is acronymous of Elton Bryson Stephens Company.
